# Treatment outcomes, safety, and characteristics of immune tolerance induction in patients with hemophilia B and inhibitors: a systematic review

**DOI:** 10.1016/j.rpth.2026.103379

**Published:** 2026-02-02

**Authors:** Shaoyu Yin, Jiahong Wu, Wen Yang, Hongli Mu, Yuexing Peng, Haoran Lu, Rong Li, Hui Bi, Zeping Zhou

**Affiliations:** Department of Hematology, The Second Affiliated Hospital of Kunming Medical University, Yunnan, China

**Keywords:** allergic reactions, hemophilia B, immune tolerance induction, nephrotic syndrome, systematic review

## Abstract

**Background:**

Inhibitor development against factor (F)IX is a serious complication in patients with hemophilia B (HB). Immune tolerance induction (ITI) aims to eliminate these inhibitors, yet variability in outcomes and treatment regimens is significant.

**Objectives:**

This systematic review synthesizes evidence on the treatment outcomes, safety, and characteristics in ITI among patients with HB and inhibitors.

**Methods:**

Following the Preferred Reporting Items for Systematic Reviews and Meta-Analysis (PRISMA) guidelines, we searched PubMed, Embase, Web of Science, and the Cochrane Library for studies of patients with congenital HB receiving ITI.

**Results:**

Eight single-arm studies involving 125 patients with HB (166 ITI attempts) were analyzed. There was significant heterogeneity in the outcome definitions and treatment protocols across studies. The success rate of ITI in patients with HB varied widely from 12.8% to 100%, with single ITI attempts showing success rates between 12.8% and 83.3%. Adverse events included allergic reactions (incidence, 0%-100%), nephrotic syndrome (incidence, 0%-33.3%), and simultaneous occurrences of both (incidence, 0%-15.4%). The median time to achieve successful immune tolerance ranged from 1.0 to 19.3 months. At least 35.9% of ITI attempts involved FIX injections combined with immunosuppressive therapy. Most ITI attempts used plasma-derived FIX, with nearly equal proportions receiving low/medium doses compared with those receiving high doses.

**Conclusions:**

The significant heterogeneity in outcome definitions and ITI protocols complicates the assessment of treatment efficacy. The high incidence of adverse events demands careful management strategies. Standardizing outcome definitions and ITI protocols is crucial to optimize patient management and improve outcomes.

## Essentials

• Patients with hemophilia B (HB) can develop factor (F)IX inhibitors, complicating their treatment.
• This review analyzed immune tolerance induction (ITI) outcomes for 125 patients with HB across studies.
• ITI success rates in patients with HB vary significantly, with a high incidence of adverse events.
• Standardizing outcome definitions and protocols is vital for improving patient care.

## 1 Introduction

Hemophilia B (HB) is an X-linked recessive bleeding disorder caused by deficiency of coagulation factor (F)IX [1]. This condition affects approximately 1 in 25,000 to 30,000 male newborns and accounts for 15% of all hemophilia cases [2]. The World Federation of Hemophilia (WFH) recommends prophylactic intravenous plasma-derived (pd) or recombinant (r)FIX product replacement therapy for patients exhibiting severe bleeding phenotypes [3]. The development of neutralizing alloantibodies (inhibitors) against FIX is the most serious complication, rendering replacement therapy ineffective and substantially increasing bleeding risk and mortality [4].

The incidence of FIX inhibitors in HB is substantially lower than that in hemophilia A (HA), occurring in approximately 1% to 5% of cases [3,[5], [6], [7], [8], [9], [10]], compared with inhibitor rates reaching 30% in HA [11,12]. For patients with HB with low-responding inhibitors, the WFH recommends using FIX-containing products to treat bleeding in the absence of allergic reactions [3]. For patients with high-responding inhibitors, recombinant activated FVII is preferred over activated prothrombin complex concentrates due to the risk of allergic reactions associated with the FIX content of activated prothrombin complex concentrates. However, prophylaxis with FIX and bypass agents in inhibitor patients is less effective and convenient than standard factor prophylaxis for those without inhibitors [13]. The primary management goal for patients with HB inhibitors is to achieve inhibitor eradication, enabling subsequent effective replacement therapy [3,14]. For HA, immune tolerance induction (ITI) represents the preferred treatment strategy for inhibitor eradication, involving repeated and regular injections of clotting factor to eliminate inhibitors [3]. ITI achieves success rates of approximately 70% to 80% in patients with HA and inhibitors [3,[15], [16], [17], [18]]. In contrast, the clearance of FIX antibodies through ITI is significantly more challenging, with reported success rates in patients with HB generally at 50% or lower [[19], [20], [21], [22]]. Moreover, while allergic reactions (ARs) and nephrotic syndrome (NS) are exceedingly rare in HA, these complications frequently accompany FIX inhibitors [3,[23], [24], [25], [26]].

Due to the rarity of HB with inhibitors and the characteristically low ITI success rates, current data on ITI in patients with HB with inhibitors derive from small cohort studies and case series. These studies exhibit significant heterogeneity in interventions and lack high-quality, evidence-based data. This knowledge gap complicates clinicians’ ability to establish rational and safe treatment regimens for patients with HB. To address this gap, we conducted a systematic review to synthesize existing evidence regarding the success rates, safety, and treatment characteristics of ITI treatment in patients with HB with inhibitors.

## 2 Methods

This systematic review follows the 2020 Preferred Reporting Items for Systematic Reviews and Meta-Analyses (PRISMA) guidelines for design and reporting [27]. The study protocol was prospectively registered and published in the International Prospective Register of Systematic Reviews (PROSPERO; registration number: CRD420251060857).

### 2.1 Study inclusion criteria

#### 2.1.1 Included studies

This systematic review included all peer-reviewed studies published as complete articless, encompassing randomized controlled trials, cohort studies, case–control studies, and cross-sectional studies. There were no publication date restrictions, and only English-language studies were included. Eligible studies were required to report ITI outcomes and safety in patients with HB.

#### 2.1.2 Participants

Studies were eligible for inclusion if they reported on adult or pediatric patients with congenital HB who had inhibitors and received ITI treatment. Any treatment approach aimed at eliminating inhibitors was considered ITI, regardless of factor product used, FIX infusion dose or frequency, or immunosuppressive therapies (including intravenous immunoglobulin, corticosteroids, cyclophosphamide, or rituximab). Studies reporting on patients with acquired HB were excluded.

#### 2.1.3 Study outcomes

Primary outcomes included ITI treatment efficacy and safety. ITI efficacy was categorized as successful (complete and partial success) or unsuccessful (failure and ongoing ITI) according to original study definitions, recognizing the absence of unified success standards in HB [14]. Safety was assessed through adverse event incidence, including bleeding, ARs, NS, and thrombosis.

Secondary outcomes included the time to successful immune tolerance achievement and treatment characteristics. The treatment characteristics cover 3 domains: (1) patient-related characteristics including ethnicity (White or non-White; African American or non–African American) [21,28] and *F9* gene mutations (high risk, large deletions and nonsense mutations; low risk, small deletions or insertions, missense mutations, or splice-site mutations) [22,29]; (2) treatment regimen–related characteristics encompassing ITI dose (low/medium dose, <100 IU/kg/d; high dose, ≥100 IU/kg/d) [19,22], type of ITI factor product (pd-FIX or rFIX) or immunosuppression (intravenous immunoglobulin, corticosteroids, cyclophosphamide, or rituximab) used [30], age at inhibitor diagnosis, age at ITI initiation, interval from inhibitor diagnosis to ITI initiation, and central venous catheter placement; and (3) inhibitor-related factors including historical peak titer (highest inhibitor titer before ITI), pre-ITI titer (most recent inhibitor titer before ITI), and peak titer during ITI (highest inhibitor titer during ITI).

### 2.2 Information sources and search strategy

Relevant studies were identified by searching 4 electronic databases: PubMed, Embase, Web of Science, and the Cochrane Library. The search strategy focused on the terms “hemophilia B” and “immune tolerance.” Complete search strategies for each database are presented in Supplementary Tables 1–4. The most recent literature search was conducted on April 17, 2025.

### 2.3 Study selection

Two authors (S.Y. and J.W.) independently screened the titles and abstracts of the retrieved studies. When titles and abstracts lacked sufficient information, the full texts were obtained for further assessment. Both researchers applied predetermined inclusion criteria to determine whether the articles should be included in the review, resolving any disagreements through discussion. In case of inconsistencies despite discussion, a third reviewer (W.Y.) was consulted.

### 2.4 Data collection and management

Duplicate publications were identified and excluded by examining author names, affiliated institutions, and study content. For studies with overlapping patient cohorts, priority was given to the most recent publication, the largest sample size, or the highest methodological quality.

Two authors independently extracted data using standardized forms. For studies with missing data, we contacted corresponding authors via email to request supplementary information. One author responded with the following data: ethnicity, *F9* genotype, age at inhibitor diagnosis or ITI initiation, interval between inhibitor diagnosis and ITI initiation, FIX product and regimen, inhibitor titers (historical peak, pre-ITI, and peak during ITI), outcomes of patients still receiving ITI at study conclusion, adverse events, and ITI treatment duration.

### 2.5 Data items

The following data were extracted from each included study: study characteristics (name of the first author, year of publication, number of persons included, country, study design, inclusion criteria, and outcome definitions); patient characteristics (age, hemophilia severity, ethnicity, and *F9* genotype); treatment characteristics; the cumulative incidence of ITI success (complete or partial); and the time to achieve successful immune tolerance, along with the cumulative incidence of adverse events during ITI.

### 2.6 Assessment of risk of bias in studies

Two authors independently assessed the risk of bias in included trials. We combined and adapted the Joanna Briggs Institute checklists for cohort and cross-sectional studies (Supplementary Table 5) (https://jbi.global/critical-appraisal-tools) to evaluate the methodological quality of each included study. Studies were classified as high quality if they met at least 11 of 13 criteria, intermediate quality if they met at least 8, and low quality if they met 7 or fewer criteria.

### 2.7 Effect measures

Continuous variables were described using median and range or mean and SD; categorical variables were described using counts and proportions. Given that some patients might have attempted multiple ITI courses, we summarized the successful cases separately by patient and by ITI attempt.

### 2.8 Data synthesis

Due to significant heterogeneity in the definitions of outcomes, ITI protocols, and treatment characteristics among the included studies, as well as the small sample sizes, we decided to conduct only a qualitative narrative synthesis instead of performing quantitative analyses.

## 3 Results

### 3.1 Study selection

The detailed study selection process is illustrated in Figure. We retrieved 1324 initial publications through electronic database searches. After removing duplicates, 2 authors reviewed the titles and abstracts of the remaining publications, identifying that 59 studies potentially met the inclusion criteria. Following a full-text review and application of the inclusion criteria, 8 studies were ultimately included. Reasons for excluding studies after full-text screening included duplicate reporting of results, failure to meet inclusion criteria, or inability to obtain full text. Supplementary Table 7 summarizes studies that initially appeared to meet inclusion criteria but were excluded upon further review.

**Figure:**
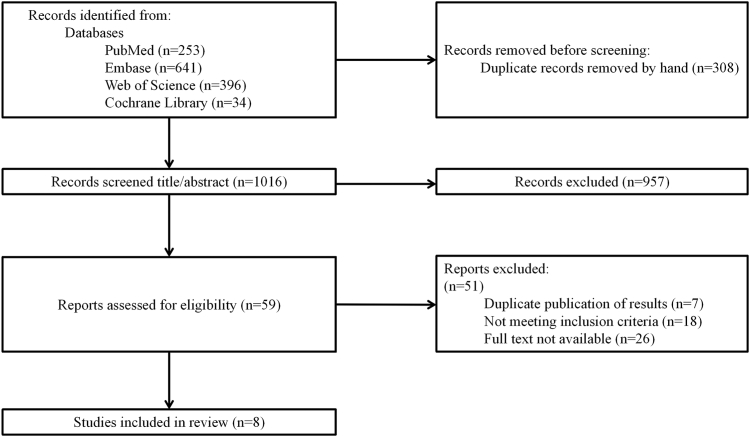
Flow chart of study selection. The literature search was conducted on April 17, 2025. After study selection, 8 studies were eligible for inclusion.

### 3.2 Included studies

Table 1 summarizes key characteristics of all included studies [9,[19], [20], [21], [22],^28^,^31^,^32^]. All 8 studies were single-arm studies. Two studies were conducted in China and 2 in Sweden; 1 study covered 4 Nordic countries (Sweden, Norway, Finland, and Denmark); and 1 was conducted in Italy, 1 across the United States and Canada; and 1 across the United States, Europe, Japan, and Australia.

**Table 1:** Study characteristics.

Study	Year	*N*	Country	Participants	Age at ITI initiation (y)	Male (%)	Severe (%)	*F9* genotype (*n*)	Definition of ITI success	Quality
Li et al. [19]	2022	12	China	Children	8.3 (1.7-17.1)	100	100	Large deletion (*n* = 8); nonsense (*n* = 4)	Inhibitor titer < 0.6 BU/mL; remaining negative thereafter	+/−
Astermark et al. [22]	2021	22	Sweden	Children and adults	3 (0.5-53)	100	100	Large deletion (*n* = 7), nonsense (*n* = 8), missense (*n* = 2), small deletion (*n* = 3), splice (*n* = 1), and complex (*n* = 1)	Inhibitor titer < 0.6 BU/mL; remaining negative thereafter	+/−
Freiburghaus et al. [32]	1999	7	Sweden	Children and adults	11.5 (5-46)	100	NR	NR	Physician judgment	+/−
Castaman et al. [9]	2013	5	Italy	Children	2 (1-12)	100	100	Nonsense (*n* = 5)	Inhibitor titer < 0.6 BU/mL; remaining negative thereafter	+/−
Dou et al. [28]	2022	13	China	Children and adults	7 (2.5-34)	100	92.3	Large deletion (*n* = 5), nonsense (*n* = 2), frameshift (*n* = 1), and splice (*n* = 1); unknown mutation type (*n* = 4)	Inhibitor titer < 0.3 BU/mL	+/−
Kihlberg et al. [20]	2022	11	Nordic countries	Children and adults	10 (1-53)	100	100	Large deletion (*n* = 4), nonsense (*n* = 3), missense (*n* = 3), and frameshift (*n* = 1)	Physician judgment (partial success as an inhibitor titer < 5 BU/mL with a response to FIX replacement therapy)	+/−
DiMichele and Kroner [21]	2002	16	United States and Canada	NR	NR	100	87.5	NR	Inhibitor titer ≤ 1 BU/mL	−
Chitlur et al. [31]	2009	39	United States, European countries, Japan, and Australia	NR	NR	100	NR	NR	Physician judgment	−

Values are reported as median (range) unless specified. Study quality was scored according to the Joanna Briggs Institute checklist as follows: +, high quality; +/−, intermediate quality; −, low quality.

BU, Bethesda Unit; F, factor; ITI, immune tolerance induction; NR, not reported.

#### 3.2.1 Study subjects

The number of subjects in each study ranged from 5 to 39. A total of 125 patients with HB with inhibitors who underwent ITI treatment were included; among them, 76 (60.8%) had severe HB, 3 (2.4%) had moderate HB, and the severity was unknown for 46 (36.8%) patients. All 8 studies involved children, with 6 studies (75%) including both adult and pediatric patients [[20], [21], [22],^28^,^31^,^32^]. Only 1 study reported the ethnic characteristics of participants [21]. Five studies (62.5%) reported the *F9* mutation types of patients [9,19,20,22,28].

#### 3.2.2 Definition of ITI success

Three studies defined complete success as two consecutive inhibitor titer measurements <0.6 Bethesda Units (BU)/mL, remaining negative thereafter [9,19,22]. One study defined success as an inhibitor titer of <0.3 BU/mL [28]. Another study defined success as an inhibitor titer of ≤1 BU/mL [21]. In 3 studies, success was determined based on physician judgment [20,31,32]. One study reporting 4 partially successful patients defined partial success as an inhibitor titer of <5 BU/mL, with a response to FIX replacement therapy [20].

### 3.3 Methodological quality of studies

When uthe modified Joanna Briggs Institute assessment tool, comprising 13 criteria for evaluating the methodological quality of included studies, was used, no study met all 13 criteria. Six studies met 10 criteria and were assessed as having intermediate quality [9,19,20,22,28,32]. The primary reason for the lower methodological quality scores was the lack of description regarding potential confounding factors or strategies to adjust for these confounders. For further details, see Supplementary Tables 5 and 6.

### 3.4 Results of individual studies

#### 3.4.1 ITI success rates

ITI success rates reported across studies varied considerably. When calculated by patient, success rates ranged from 12.8% to 100%. When calculated by ITI attempt, success rates ranged from 12.8% to 83.3%, with results from each study found in Table 2. Of 23 ITI attempts that involved allergic reactions, 6 were successful [19,20,22,28].

**Table 2:** Treatment characteristics for ITI.

Study	*N*	ITI attempts	ITI regimen (*n*^a^)	ITI product (*n*^a^)			Immunosuppression	Interval—ITI start (mo)	Pre-ITI titer (BU/mL)	Historical peak titer (BU/mL)	Time to achieve successful immune tolerance (mo)	ITI outcomes, *n* (%)		ITI safety, *n* (%)^a^		
				pd	r	Unknown						Patients with successful ITI	Successful ITI attempts	AR	NS	AR and NS
Li et al. [19]	12	16	Low/medium dose (*n* = 16) High dose (*n* = 0)	16	0	0	16/16	26.4 (1.2-103.2)	16.3 (5-256)	54.2 (6.8-512)	1.8 (0.9-43.3)	6 (50)	8 (50)	5 (31.3)	2 (12.5)	1 (6.3)
Astermark et al. [22]	22	40	Low/medium dose (*n* = 9) High dose (26) Unknown (*n* = 5)	21	18	1	34/40	9.6 (0-324)	0.6 (0.2-65)	4.2 (0.5-1380)	9.5 (0.5-26.5)	11 (50)	13 (32.5)	13 (32.5)	6 (15)	3 (7.5)
Freiburghaus et al. [32]	7	14	Low/medium dose (*n* = 3) High dose (*n* = 11)	14	0	0	14/14	84 (36-288)	9.8 (0.3-159)	303 (0.9-603)	1.0 (0.5-1.93)	6 (85.7)	6 (42.9)	0 (0)	0 (0)	0 (0)
Castaman et al. [9]	5	6	Low/medium dose (*n* = 5) High dose (*n* = 1)	2	3	1	0/6	NR	NR	16 (2.6-117)	5.0 (0.2-52.5)	5 (100)	5 (83.3)	0 (0)	0 (0)	0 (0)
Dou et al. [28]	13	13	Low/medium dose (*n* = 11) High dose (*n* = 0) Unknown (*n* = 2)	13	0	0	12/13	24 (0-120)	15 (5.2-167)	98 (5.2-230)	17.0 (12.0-20.0)	3 (23.1)	3 (23.1)	4 (30.1)	2 (15.4)	2 (15.4)
Kihlberg et al. [20]	11	22	Low/medium dose (*n* = 6) High dose (*n* = 10) Unknown (*n* = 6)	18	4	0	16/22	24 (0-444)	2.1 (0.2-150)	40 (0.9-305)	19.3 (0.8-99.0)	8 (72.7)^b^	8 (36.4)	9 (40.1)	3 (13.6)	3 (13.6)
DiMichele and Kroner [21]	16	16	NR	NR	NR	NR	14/39	NR	1-24	2.4-650	1 (0.4-1.5)	5 (31.3)	5 (31.3)	11 (68.8)	3 (18.8)	NR
Chitlur et al. [31]	39	39	NR	NR	NR	NR	8/16	44.1 (0-227)	NR	30 (1-1156)	NR	5 (12.8)	5 (12.8)	39 (100)	13 (33.3)	NR

Numbers are reported as median (range), except for the pre-ITI titer and historical peak titer in the study by DiMichele and Kroner [21], which are reported only as ranges. Low/medium dose, <100 IU/kg/d; high dose, ≥100 IU/kg/d.

AR, allergic reaction; BU, Bethesda Unit; F, factor; ITI, immune tolerance induction; NR, not reported; NS, nephrotic syndrome; pd, plasma-derived coagulation FIX; r, recombinant coagulation FIX.

a ITI attempts.

b Four patients had partial success.

#### 3.4.2 Adverse events

Among the adverse events reported in the studies, the rates of ARs ranged from 0% to 100%, while NS rates varied from 0% to 33.3%. The combined rates of ARs and NS ranged from 0% to 15.4%. Only 1 study reported bleeding events [19], another study reported adverse events related to central venous catheters [21], and no studies reported instances of thrombosis.

#### 3.4.3 Time to successful immune tolerance achievement

The median time to achieve successful immune tolerance ranged from 1.0 to 19.3 months (Table 2).

#### 3.4.4 ITI treatment characteristics

Six studies described the types of FIX products and dosing regimens used in ITI treatment [9,19,20,22,28,32]. A total of 125 patients underwent 166 ITI attempts (with 1 ITI attempt equal to 1 course of ITI), including 50 patients receiving 76 ITI treatments using only pd-FIX, 14 patients receiving 17 ITI treatments using only rFIX, and 6 patients sequentially using both pd-FIX and rFIX for 16 ITI attempts. Additionally, there were 57 ITI attempts using unknown factor types. Thirty-four patients received only low/medium-dose ITI (40 attempts total), 24 patients received only high-dose ITI (36 attempts total), and 7 patients received a combination of these regimens (21 attempts total), with the dose unknown for 69 ITI attempts. Except for 1 study [9], the remaining studies used immunosuppressive therapy to varying degrees, with usage rates, ranging from 35.9% to 100%. The interval details from inhibitor diagnosis to ITI initiation, as well as historical peak titer and pre-ITI titer, can be found in Table 2. The treatment characteristics corresponding to the ITI outcomes can be found in Supplementary Table 8.

## 4 Discussion

This systematic review summarized data from 8 studies involving 125 patients with HB with inhibitors, providing the most comprehensive evidence to date for this rare clinical condition. Currently, research on ITI for patients with HB reveals considerable heterogeneity in both the definitions of success and treatment protocols, along with small sample sizes. Our findings indicate that the success rate of ITI in patients with HB ranges from 12.8% to 100%, with the success rate for single ITI attempts varying between 12.8% and 83.3%. For patients experiencing ARs during ITI, the success rate varied from 0% to 50%. Throughout the single ITI attempts, the incidence rates of ARs and NS and the simultaneous occurrence of both were recorded at 0% to 100%, 0% to 33.3%, and 0% to 15.4%, respectively. The median time to achieve successful immune tolerance ranged from 1.0 to 19.3 months. Seven studies reported that at least 35.9% of patients received FIX injections combined with immunosuppressive therapy as part of their ITI regimen. Most patients underwent ITI using pd-FIX, with nearly equal proportions of patients receiving low/medium doses versus high doses for ITI.

The low incidence of inhibitors in patients with HB [3,[5], [6], [7], [8], [9], [10]], coupled with the potential for severe complications during ITI [3,[23], [24], [25], [26]], poses significant challenges to conducting high-quality, large-scale clinical studies. In the studies we reviewed, some used thresholds of 0.3, 0.6, and 1 BU/mL to determine ITI success, while others relied on subjective clinical judgment for assessment. There are considerable discrepancies in how success is defined across these studies. Moreover, the treatment protocols used exhibit substantial variation. Currently, unlike patients with HA, there is no established standardized ITI treatment protocol for patients with HB. Aside from the study by Li et al. [19], existing research primarily depends on the empirical practices of various treatment centers, resulting in patients at the same center potentially receiving different treatment regimens; in fact, even the same patient may undergo varying treatment approaches across multiple ITI courses. Additionally, the sample sizes in current studies are generally small. While the study by Li et al. [19] defined ITI success based on inhibitor titers and used a uniform treatment schedule for all patients, the small sample size also affected the robustness of the findings. The significant heterogeneity in the definitions of ITI success and treatment protocols, combined with small sample sizes, has resulted in substantial variations in the reported success rates of ITI in patients with HB. This inconsistency not only severely undermines the interpretability of the results but also affects their accuracy. The absence of these standards is currently the primary issue that needs to be addressed.

In more than half of the studies, the success rate of ITI in patients with HB (range, 12.8%-50%) is lower than that of patients with HA (range, 70%-80%) [3,[15], [16], [17], [18]]. This phenomenon may be related to the higher incidence of adverse events during ITI in patients with HB and the differences in potential immune response patterns between the 2 diseases. In 2 studies with higher success rates, the success of ITI often depends on the subjective judgment of the physician, which may lead to potential publication bias. Additionally, these 2 studies have the smallest sample sizes, resulting in poor robustness of the findings. Some patients in the studies underwent multiple attempts at ITI, with some still achieving immune tolerance, suggesting that this treatment strategy should not be entirely abandoned after an initial failure of ITI [20,28]. Notably, with close monitoring and proactive management, some patients who experience ARs may still achieve immune tolerance [19,20,22,28]. In the included studies, only 1 patient with NS achieved tolerance, and multiple studies indicate that NS is a significant factor contributing to the failure of ITI treatment in most patients [12,18,20,22,33], with kidney disease potentially being irreversible [3,34]. Research suggests NS is associated with ARs both before and during ITI [31,34]. These patients need to carefully weigh the benefits against the risks; for patients who experience ARs before or during treatment, regular urinalysis should be performed to monitor for proteinuria to identify early NS [35].

In this study, although most researchers actively adopted a strategy of combining FIX with immunosuppressants to prevent ARs, the incidence of ARs remained high. This may indicate that, despite aggressive immunosuppressive measures, AR remains a difficult complication to avoid during ITI. To address the occurrence of ARs, some studies have proposed desensitization therapy through repeated intravenous or subcutaneous administration of small doses of FIX before initiating ITI to reduce the risk of ARs [23,[36], [37], [38], [39]]. However, WFH guidelines suggest this approach may exacerbate ARs or lead to anaphylactic shock [3], so it should be attempted with caution. Although the incidence of NS is lower than that of ARs, combined immunosuppressive therapy does not completely prevent the occurrence of NS. The WFH guidelines state that high doses of FIX may increase the risk of NS [3]; therefore, low to moderate doses of FIX could be considered for ITI to minimize the occurrence of NS. Only 1 study reported bleeding events during ITI, indicating that bleeding during ITI treatment was reduced compared with before treatment [19]. Additionally, only 1 study highlighted complications related to central venous catheters implanted during ITI, suggesting the potential for higher rates of central venous catheter–related complications in the induction failure group; however, the causal relationship between the 2 remains unclear [21].

The median time for patients with HB to successfully achieve immune tolerance varies significantly, which again reflects the substantial differences in the definition of ITI success and treatment protocols for patients with HB. Notably, Freiburghaus et al. [32] used immunoadsorption therapy prior to initiating ITI, and in the study by Freiburghaus et al. [32], the speed of tolerance achievement in patients was significantly faster than that in other studies, suggesting that immunoadsorption may shorten the duration of ITI.

This study found that the incidence of ARs and NS in patients with HB is significantly higher than that in patients with HA, leading to a significantly higher proportion of patients with HB receiving immunosuppressive therapy than that of patients with HA [3]. Most patients underwent ITI using pd-FIX, with nearly equal proportions receiving low/medium doses versus high doses. However, the considerable differences in the definitions of ITI success and treatment protocols for patients with HB complicate the reliable assessment of the efficacy of various factor concentrates, dosages, and immunosuppressive regimens. Additionally, the significant heterogeneity in success definitions and treatment approaches, combined with small sample sizes, hinders the ability to accurately evaluate the effects of patient-related and inhibitor-related factors on ITI outcomes and the occurrence of adverse events.

Our systematic review indicates that the current ITI treatment for patients with HB lacks a consistent definition of success and standardized treatment protocols. This has resulted in significant variations in ITI success rates across studies. Although immunosuppressive therapy is actively pursued in ITI, it is associated with a high incidence of adverse events. There is an urgent need to establish standardized international prospective registry systems for patients with HB with inhibitors, using uniform definitions of success and treatment protocols to obtain higher quality evidence for better guidance of clinical practice.
