# Supplementary material for: Treatment outcomes, safety, and characteristics of immune tolerance induction in patients with hemophilia B and inhibitors: a systematic review
Source: Res Pract Thromb Haemost. 2026 Feb 2;10(2):103379. doi: 10.1016/j.rpth.2026.103379 (PMC13015575; doi:10.1016/j.rpth.2026.103379)
Supplement: Supplementary Material [file mmc1.docx]

**Supplementary Table S1. PubMed Search Strategy (April 17, 2025)**

| # | Search Strategy | Results |
| --- | --- | --- |
| 1 | ("Hemophilia B"[Mesh]) OR "Factor IX"[Mesh] | 8,512 |
| 2 | (("hemophilia B"[Title/Abstract]) OR ("Haemophilia B"[Title/Abstract])) OR (("factor 9"[Title/Abstract] OR "factor IX"[Title/Abstract] OR F9[Title/Abstract]) AND (deficienc*[Title/Abstract] OR disorder*[Title/Abstract])) | 4,205 |
| 3 | (("Hemophilia B"[Mesh]) OR "Factor IX"[Mesh]) OR ((("hemophilia B"[Title/Abstract]) OR ("Haemophilia B"[Title/Abstract])) OR (("factor 9"[Title/Abstract] OR "factor IX"[Title/Abstract] OR F9[Title/Abstract]) AND (deficienc*[Title/Abstract] OR disorder*[Title/Abstract]))) | 9,896 |
| 5 | "Immune Tolerance"[Mesh] | 47,542 |
| 8 | (((((Immune Tolerance[Title/Abstract]) OR (immunotolerance[Title/Abstract])) OR (Tolerance, Immune[Title/Abstract])) OR (ITI[Title/Abstract])) OR (Immunosuppress*[Title/Abstract])) OR (Immunomodulat*[Title/Abstract]) | 299,001 |
| 9 | (bonn[Title/Abstract] OR creveld[Title/Abstract] OR malmo[Title/Abstract]) | 4,765 |
| 11 | ((rituximab[Title/Abstract] OR cyclophosphamide[Title/Abstract] OR prednisone[Title/Abstract] OR immunoglobulins, intravenous[Title/Abstract] OR "mtor inhibitors"[Title/Abstract] OR anti-cd20[Title/Abstract] OR "intravenous immunoglobulins"[Title/Abstract] OR "mycophenolate mofetil"[Title/Abstract] OR ivig[Title/Abstract] OR sirolimus[Title/Abstract] OR rapamycin[Title/Abstract] OR rapalogs[Title/Abstract] OR "cd20 antibody"[Title/Abstract]) OR glucocorticoids[Title/Abstract] OR dexamethasone[Title/Abstract] OR methylprednisolone[Title/Abstract] OR hydrocortisone[Title/Abstract]) | 310,690 |
| 12 | ((("Immune Tolerance"[Mesh]) OR ((((((Immune Tolerance[Title/Abstract]) OR (immunotolerance[Title/Abstract])) OR (Tolerance, Immune[Title/Abstract])) OR (ITI[Title/Abstract])) OR (Immunosuppress*[Title/Abstract])) OR (Immunomodulat*[Title/Abstract]))) OR ((bonn[Title/Abstract] OR creveld[Title/Abstract] OR malmo[Title/Abstract]))) OR (((rituximab[Title/Abstract] OR cyclophosphamide[Title/Abstract] OR prednisone[Title/Abstract] OR immunoglobulins, intravenous[Title/Abstract] OR "mtor inhibitors"[Title/Abstract] OR anti-cd20[Title/Abstract] OR "intravenous immunoglobulins"[Title/Abstract] OR "mycophenolate mofetil"[Title/Abstract] OR ivig[Title/Abstract] OR sirolimus[Title/Abstract] OR rapamycin[Title/Abstract] OR rapalogs[Title/Abstract] OR "cd20 antibody"[Title/Abstract]) OR glucocorticoids[Title/Abstract] OR dexamethasone[Title/Abstract] OR methylprednisolone[Title/Abstract] OR hydrocortisone[Title/Abstract])) | 608,970 |
| 13 | ((("Hemophilia B"[Mesh]) OR "Factor IX"[Mesh]) OR ((("hemophilia B"[Title/Abstract]) OR ("Haemophilia B"[Title/Abstract])) OR (("factor 9"[Title/Abstract] OR "factor IX"[Title/Abstract] OR F9[Title/Abstract]) AND (deficienc*[Title/Abstract] OR disorder*[Title/Abstract])))) AND (((("Immune Tolerance"[Mesh]) OR ((((((Immune Tolerance[Title/Abstract]) OR (immunotolerance[Title/Abstract])) OR (Tolerance, Immune[Title/Abstract])) OR (ITI[Title/Abstract])) OR (Immunosuppress*[Title/Abstract])) OR (Immunomodulat*[Title/Abstract]))) OR ((bonn[Title/Abstract] OR creveld[Title/Abstract] OR malmo[Title/Abstract]))) OR (((rituximab[Title/Abstract] OR cyclophosphamide[Title/Abstract] OR prednisone[Title/Abstract] OR immunoglobulins, intravenous[Title/Abstract] OR "mtor inhibitors"[Title/Abstract] OR anti-cd20[Title/Abstract] OR "intravenous immunoglobulins"[Title/Abstract] OR "mycophenolate mofetil"[Title/Abstract] OR ivig[Title/Abstract] OR sirolimus[Title/Abstract] OR rapamycin[Title/Abstract] OR rapalogs[Title/Abstract] OR "cd20 antibody"[Title/Abstract]) OR glucocorticoids[Title/Abstract] OR dexamethasone[Title/Abstract] OR methylprednisolone[Title/Abstract] OR hydrocortisone[Title/Abstract]))) | 463 |
| 14 | (("Review" [Publication Type]) OR "Case Reports" [Publication Type]) OR "Meta-Analysis" [Publication Type] | 6,017,934 |
| 15 | (((("Hemophilia B"[Mesh]) OR "Factor IX"[Mesh]) OR ((("hemophilia B"[Title/Abstract]) OR ("Haemophilia B"[Title/Abstract])) OR (("factor 9"[Title/Abstract] OR "factor IX"[Title/Abstract] OR F9[Title/Abstract]) AND (deficienc*[Title/Abstract] OR disorder*[Title/Abstract])))) AND (((("Immune Tolerance"[Mesh]) OR ((((((Immune Tolerance[Title/Abstract]) OR (immunotolerance[Title/Abstract])) OR (Tolerance, Immune[Title/Abstract])) OR (ITI[Title/Abstract])) OR (Immunosuppress*[Title/Abstract])) OR (Immunomodulat*[Title/Abstract]))) OR ((bonn[Title/Abstract] OR creveld[Title/Abstract] OR malmo[Title/Abstract]))) OR (((rituximab[Title/Abstract] OR cyclophosphamide[Title/Abstract] OR prednisone[Title/Abstract] OR immunoglobulins, intravenous[Title/Abstract] OR "mtor inhibitors"[Title/Abstract] OR anti-cd20[Title/Abstract] OR "intravenous immunoglobulins"[Title/Abstract] OR "mycophenolate mofetil"[Title/Abstract] OR ivig[Title/Abstract] OR sirolimus[Title/Abstract] OR rapamycin[Title/Abstract] OR rapalogs[Title/Abstract] OR "cd20 antibody"[Title/Abstract]) OR glucocorticoids[Title/Abstract] OR dexamethasone[Title/Abstract] OR methylprednisolone[Title/Abstract] OR hydrocortisone[Title/Abstract])))) NOT ((("Review" [Publication Type]) OR "Case Reports" [Publication Type]) OR "Meta-Analysis" [Publication Type]) | 253 |

**Supplementary Table S2. Embase Search Strategy (April 17, 2025)**

| # | Search strategy | Results |
| --- | --- | --- |
| #1 | 'hemophilia b'/exp OR 'blood clotting factor 9'/exp | 17714 |
| #2 | 'hemophilia b':ti,ab,kw OR 'haemophilia b':ti,ab,kw OR (('factor 9':ti,ab,kw OR 'factor ix':ti,ab,kw OR 'f9':ti,ab,kw) AND ('deficienc*':ti,ab,kw OR 'disorder*':ti,ab,kw)) | 7958 |
| #3 | #1 OR #2 | 19014 |
| #4 | 'immunological tolerance'/exp | 59070 |
| #5 | 'immunological tolerance':ti,ab,kw OR 'immune tolerance':ti,ab,kw OR 'immunotolerance':ti,ab,kw OR 'tolerance, immune':ti,ab,kw OR 'iti':ti,ab,kw OR 'immunosuppress*':ti,ab,kw OR 'immunomodulat*':ti,ab,kw | 466428 |
| #6 | 'bonn':ti,ab,kw OR 'creveld':ti,ab,kw OR 'malmo':ti,ab,kw | 6531 |
| #7 | 'rituximab':ti,ab,kw OR 'cyclophosphamide':ti,ab,kw OR 'prednisone':ti,ab,kw OR 'immunoglobulins, intravenous':ti,ab,kw OR 'mtor inhibitors':ti,ab,kw OR 'anti-cd20':ti,ab,kw OR 'intravenous immunoglobulins':ti,ab,kw OR 'mycophenolate mofetil':ti,ab,kw OR 'ivig':ti,ab,kw OR 'sirolimus':ti,ab,kw OR 'rapamycin':ti,ab,kw OR 'rapalogs':ti,ab,kw OR 'cd20 antibody':ti,ab,kw | 315877 |
| #8 | 'rituximab':ti,ab,kw OR 'cyclophosphamide':ti,ab,kw OR 'prednisone':ti,ab,kw OR 'immunoglobulins, intravenous':ti,ab,kw OR 'mtor inhibitors':ti,ab,kw OR 'anti-cd20':ti,ab,kw OR 'intravenous immunoglobulins':ti,ab,kw OR 'mycophenolate mofetil':ti,ab,kw OR 'ivig':ti,ab,kw OR 'sirolimus':ti,ab,kw OR 'rapamycin':ti,ab,kw OR 'rapalogs':ti,ab,kw OR 'cd20 antibody':ti,ab,kw OR 'glucocorticoids':ti,ab,kw OR 'dexamethasone':ti,ab,kw OR 'methylprednisolone':ti,ab,kw OR 'hydrocortisone':ti,ab,kw | 500651 |
| #9 | #4 OR #5 OR #6 OR #7 OR #8 | 934445 |
| #10 | #3 AND #9 | 1176 |
| #11 | 'case report'/exp OR 'review'/exp OR 'meta analysis'/exp | 6704166 |
| #12 | #10 NOT #11 | 781 |
| #13 | #12 AND 'human'/de | 641 |

**Supplementary Table S3. Web of science Search Strategy (April 17, 2025)**

| **#** | **Search strategy** | **Results** |
| --- | --- | --- |
| 1 | (TS=(hemophilia B)) OR TS=(haemophilia B) and Preprint Citation Index (Exclude – Database) | 16135 |
| 2 | (((TS=("hemophilia b")) OR TS=("haemophilia b")) OR TS=(("factor 9" OR "factor IX" OR F9) NEAR (deficienc* OR disorder*))) and Preprint Citation Index (Exclude – Database) | 10817 |
| 3 | #2 OR #1 and Preprint Citation Index (Exclude – Database) | 16508 |
| 4 | TS=(Immune Tolerance) and Preprint Citation Index (Exclude – Database) | 125431 |
| 5 | (((((TS=("Immune Tolerance" )) OR TS=(immunotolerance)) OR TS=(Tolerance, Immune)) OR TS=(ITI)) OR TS=(Immunosuppress*)) OR TS=(Immunomodulat*) and Preprint Citation Index (Exclude – Database) | 674138 |
| 6 | TS=(rituximab OR cyclophosphamide OR prednisone OR immunoglobulins, intravenous OR "mtor inhibitors" OR anti-cd20 OR "intravenous immunoglobulins" OR "mycophenolate mofetil" OR ivig OR sirolimus OR rapamycin OR rapalogs OR "cd20 antibody" OR glucocorticoids OR dexamethasone OR methylprednisolone OR hydrocortisone) and Preprint Citation Index (Exclude – Database) | 727575 |
| 7 | ((TS=(bonn)) OR TS=(creveld)) OR TS=(malmo) and Preprint Citation Index (Exclude – Database) | 11801 |
| 8 | #4 OR #5 OR #6 OR #7 and Preprint Citation Index (Exclude – Database) | 1283426 |
| 9 | #3 AND #8 and Preprint Citation Index (Exclude – Database) | 1528 |
| 10 | #3 AND #8 and Preprint Citation Index (Exclude – Database) and Review Article (Exclude – Document Types) | 1168 |
| 11 | #3 AND #8 and Preprint Citation Index (Exclude – Database) and Review Article (Exclude – Document Types) and Meeting or Case Report (Exclude – Document Types) | 718 |
| 12 | #3 AND #8 and Preprint Citation Index (Exclude – Database) and Review Article (Exclude – Document Types) and Meeting or Case Report (Exclude – Document Types) and English (Languages) | 691 |
| 13 | #3 AND #8 and Preprint Citation Index (Exclude – Database) and Review Article (Exclude – Document Types) and Meeting or Case Report (Exclude – Document Types) and English (Languages) and Humans (MeSH Headings) | 396 |

**Supplementary Table S4. Cochrane Library Search Strategy (April 17, 2025)**

| # | Search strategy | Results |
| --- | --- | --- |
| #1 | MeSH descriptor: [Hemophilia B] explode all trees | 168 |
| #2 | MeSH descriptor: [Factor IX] explode all trees | 111 |
| #3 | #1 OR #2 | 220 |
| #4 | (hemophilia B):ti,ab,kw OR (Haemophilia B):ti,ab,kw OR (("factor 9" or "factor IX" or F9) AND (deficienc* OR disorder*)):ti,ab,kw | 746 |
| #5 | #3 OR #4 | 789 |
| #6 | MeSH descriptor: [Immune Tolerance] explode all trees | 540 |
| #7 | ("Immune Tolerance"):ti,ab,kw OR (immunotolerance):ti,ab,kw OR (Tolerance, Immune):ti,ab,kw OR (ITI):ti,ab,kw OR(Immunosuppress*):ti,ab,kw OR(Immunomodulat*):ti,ab,kw | 26395 |
| #8 | (bonn or creveld or malmo):ti,ab,kw | 318 |
| #9 | (rituximab):ti,ab,kw OR (cyclophosphamide):ti,ab,kw OR (prednisone):ti,ab,kw OR (immunoglobulins, intravenous):ti,ab,kw OR ("mtor inhibitors"):ti,ab,kw OR (anti-cd20):ti,ab,kw OR ("intravenous immunoglobulins"):ti,ab,kw OR ("mycophenolate mofetil"):ti,ab,kw OR (ivig):ti,ab,kw OR (sirolimus):ti,ab,kw OR (rapamycin):ti,ab,kw OR (rapalogs):ti,ab,kw OR ("cd20 antibody"):ti,ab,kw OR (glucocorticoids):ti,ab,kw OR (dexamethasone):ti,ab,kw OR (methylprednisolone):ti,ab,kw OR (Hydrocortisone):ti,ab,kw | 69545 |
| #10 | #6 OR #7 OR #8 OR #9 | 88717 |
| #11 | #5 AND #10 | 37 |

**Supplementary Table S5. Quality Assessment.** JBI Critical Appraisal checklist for cohort- and cross-sectional studies

| **Reviewer** |  |
| --- | --- |
| **Date** |  |
| **Title** |  |
| **Author** |  |
| **Year** |  |

|  | Yes | No | Unclear | Not applicable |
| --- | --- | --- | --- | --- |
| 1. **Were the criteria for inclusion in the sample clearly defined?**  - Yes: clear description of the included population, e.g. type of hemophilia, acquired or congenital hemophilia, severity of hemophilia. | ☐ | ☐ | ☐ | ☐ |
| 1. **Were the study subjects described in detail?**  - Yes: e.g. if demographic data, such as sex, ethnicity/descent or age; or *F9* genotype or hemophilia severity were described (≥2 = yes). | ☐ | ☐ | ☐ | ☐ |
| 1. **Was the setting described in detail?**  - Yes: if where was clearly described; f.e. if the country or name of the hemophilia treatment center were described. | ☐ | ☐ | ☐ | ☐ |
| 1. **Was the setting described in detail?**  - Yes: if when was clearly described; f.e. if the time period of the study described. | ☐ | ☐ | ☐ | ☐ |
| 1. **Was the immune tolerance induction clearly described?**  - Yes: if the provide on information on the immune tolerance induction regimen. | ☐ | ☐ | ☐ | ☐ |
| 1. **Were the outcome measurements objectively defined?**  - Yes: clear definition of the outcome measurements, used in every subject. | ☐ | ☐ | ☐ | ☐ |
| 1. **Were confounding factors identified?**  - Yes: if other factors than the determinant that could potentially attribute to ITI outcome, were clearly described. | ☐ | ☐ | ☐ | ☐ |
| 1. **Were strategies to deal with confounding factors stated?**  - Yes: if strategies to adjust for confounding factors for ITI outcome were clearly described in the methods. (NA if question 7 is answered no). | ☐ | ☐ | ☐ | ☐ |
| 1. **Were the determinants for ITI outcome measured in a valid and reliable way?**  - Yes: if the determinants (e.g. peak titer, pre-ITI titer) were measured or evaluated in all subjects. | ☐ | ☐ | ☐ | ☐ |
| 1. **Was the follow up time reported and long enough for outcomes to occur?**  - Yes: if the follow up time was reported and if ITI success or failure could be achieved. | ☐ | ☐ | ☐ | ☐ |
| 1. **Was follow up complete, and if not, were the reasons to loss to follow up described and explored?**  - Yes: if all persons reached the ITI endpoint, or description of patients with ongoing ITI or if reasons to loss to follow up were described. | ☐ | ☐ | ☐ | ☐ |
| 1. **Were strategies to address incomplete follow up utilized?**  - Yes: if a strategy is described to address incomplete follow-up. (NA if question 11 is answered yes). | ☐ | ☐ | ☐ | ☐ |
| 1. **Was statistical analysis appropriate?**  - Yes: if the statistical analysis was appropriate for the population to calculate the outcome measure, if odds ratios or relative risk ratios were calculated with corresponding confidence intervals. | ☐ | ☐ | ☐ | ☐ |
| **TOTAL** | **Yes** | **No** | **Unclear** | **NA** |
|  |  |  |  |  |

**Supplementary Table S6.** **Methodological quality assessment.** Results methodological quality of each included study

| Study | **Q1** | **Q2** | **Q3** | **Q4** | **Q5** | **Q6** | **Q7** | **Q8** | **Q9** | **Q10** | **Q11** | **Q12** | **Q13** | TOTAL |
| --- | --- | --- | --- | --- | --- | --- | --- | --- | --- | --- | --- | --- | --- | --- |
|  | Target population | Subject descriptives | Setting where | Setting when | Therapy definitions | Outcome definitions | Confounding | Strategies confounding | Outcome measurements | Follow-up | Loss of follow-up | Strategies incomplete follow-up | Statistical analysis |  |
| Li[19] | Y | Y | Y | Y | Y | Y | N | NA | Y | Y | Y | NA | Y | 10 |
| Astermark[22] | Y | Y | Y | Y | Y | Y | N | NA | Y | Y | Y | NA | Y | 10 |
| Freiburghaus[41] | Y | N | Y | Y | Y | Y | Y | N | Y | Y | Y | NA | Y | 10 |
| Castaman[11] | Y | Y | Y | Y | Y | Y | N | NA | Y | Y | Y | NA | Y | 10 |
| Dou[33] | Y | Y | Y | Y | Y | Y | N | NA | Y | Y | Y | NA | Y | 10 |
| Kihlberg[20] | Y | Y | Y | Y | Y | Y | N | NA | Y | Y | Y | NA | Y | 10 |
| DiMichele[21] | Y | N | Y | Y | N | N | N | NA | N | Y | Y | NA | Y | 6 |
| Chitlur[29] | Y | N | Y | Y | Y | N | N | NA | N | N | Y | NA | Y | 6 |

Abbreviations: Y = yes, N = no, NA = not applicable, U = unknown

|  | 11-13 | High-quality |
| --- | --- | --- |
|  | 8-10 | Intermediate-quality |
|  | 0-7 | Low-quality |

**Supplementary Table S7. Reason for exclusion of studies**

| Title | Author | Year | Reason for exclusion |
| --- | --- | --- | --- |
| Immune tolerance induction for fix inhibitors using combined B and T cell immune modulation therapy in severe hemophilia b | Abajas | 2018 | Full text not available |
| Inhibitors incidence in the Colombian population with hemophilia | Alvis | 2018 | Full text not available |
| Current European practice in immune tolerance induction therapy in patients with haemophilia and inhibitors | Astermark | 2006 | guidelines |
| Malmo International Brother Study (MIBS): An international survey of brother pairs with haemophilia | Astermark | 1999 | Full text not available |
| Haemophilia in Spain | Aznar | 2009 | The article does not involve the content of inhibitor treatment |
| Clinical Characteristics, Therapy and Outcome of Children with Hemophilia B and Inhibitors: a PedNet Study | Barg | 2023 | Full text not available |
| The international factor IX treatment network survey | Berntorp | 2012 | The article does not involve the content of inhibitor treatment |
| Immune tolerance induction and the treatment of hemophilia. Malmö protocol update. | Berntorp | 2000 | Full text not available |
| Malmo protocol update | Berntorp | 2000 | Full text not available |
| Haemophilia care and outcome in a major haemophilia treatment centre in Malaysia | Boo | 2021 | The main focus of the study is prophylactic treatment |
| Clinical review of patients with haemophilia (PWH) in a Malaysian tertiary center | Boo | 2020 | Full text not available |
| 35 years of immune tolerance treatment - Bonn protocol | Brackmann | 2011 | Full text not available |
| The occurrence of immunological tolerance to inhibitors of factors VIII and IX in hemophilia patients | Brakman | 1993 | Non-English article |
| An International Prophylaxis Study Group (IPSG) survey of prophylaxis in inhibitor positive children/adults with severe haemophilia | Carcao | 2017 | guidelines |
| Inhibitors in Italian hemophilia B patients | Castaman | 2012 | Full text not available |
| Trends in hemophilia clinician prescribing practices: 1999 and 2015 | Curtis | 2007 | Full text not available |
| Immune tolerance for haemophilia patients with inhibitors: Analysis of the western United States experience | Damiano | 2000 | The main research content of the article is the treatment of hemophilia A with inhibitors |
| The maintenence of tolerance after successful immune tolerance induction in hemophilia A and B: The North American registry | DiMichele | 2000 | Full text not available |
| The North American Immune Tolerance Registry: Contributions to the thirty-year experience with immune tolerance therapy | DiMichele | 2009 | Duplicate publication of cohort DiMichele et al. (2002)  The North American Immune Tolerance Registry: Practices, Outcomes, Outcome Predictors |
| Analysis of the North American Immune Tolerance Registry (NAITR) 1993-1997: current practice implications. ISTH Factor VIII/IX Subcommittee Members | DiMichele | 1999 | Duplicate publication of cohort DiMichele et al. (2002)  The North American Immune Tolerance Registry: Practices, Outcomes, Outcome Predictors |
| Patients with hemophilia B with inhibitors in China | Dou | 2021 | Duplicate publication of cohort Dou et al. (2023)  Factor IX inhibitors in haemophilia B: A report of National Haemophilia Registry in China |
| Immunoadsorption for removal of inhibitors: update on treatments in Malm?Lund between 1980 and 1995. | Freiburghaus | 1998 | Duplicate publication of cohort Freiburghaus et al. (1999)  Tolerance induction using the Malmo treatment model 1982-1995 |
| Allergic reaction after replacement therapy in haemophilia B paediatric patient with inhibitor to factor IX | Giordano | 2011 | Full text not available |
| The new era of Haemophilia in a Comprehensive Hemophilia Treatment Center in Mexico | Gonzalez | 2022 | Full text not available |
| 10 Years comparative hemophilia prophylaxis across São Paulo State-Brazil | Guersoni | 2022 | Full text not available |
| Immune tolerance treatment in haemophilia patients with inhibitors: the Spanish Registry | Haya | 2001 | The main research content of the article is the predictors of inhibitors in hemophilia A |
| Current view and outcome of ITI therapy - A change over time? | Holstein | 2016 | The main research content of the article is the treatment of hemophilia A with inhibitors |
| Inhibitor eradication in haemophilia: An European survey | Holstein | 2015 | Full text not available |
| Inhibitor eradication in hemophilia: A European survey | Holstein | 2014 | Full text not available |
| Improvement in anti-hemophilic preparations and its problems. 3. Treatment of hemophilia patients with inhibitors; induction of immune tolerance by a low or intermediate dose regimen | Iizuka | 1988 | Non-English article |
| Inhibitor eradication in hemophilia: A European survey | Kiehm | 2014 | Full text not available |
| Successful immunotolerance induction in two boys with haemophilia B and inhibitor | Klukowska | 2011 | Full text not available |
| Inhibitor problem and treatment of bleeding episodes in a hemophilia B patient | Kupesiz | 2011 | Full text not available |
| The German Registry of immune tolerance treatment in hemophilia--1999 update | Lenk | 2000 | Full text not available |
| Successful immune tolerance induction in two Polish boys with haemophilia B and inhibitors | Lenk | 1999 | Case Report |
| Eradication of FIX inhibitor in haemophilia B children: A retrospective study from haemophilia pediatric comprehensive care center of China | Li | 2022 | Duplicate publication of cohort Li et al. (2022)  Eradication of FIX inhibitor in haemophilia B children using low-dose immune tolerance induction with rituximab-based immunosuppressive agent(s) in China |
| Current status of haemophilia inhibitor management in mainland China: a haemophilia treatment centres survey on treatment preferences and real-world clinical practices | Liu | 2021 | The primary purpose of the study is to investigate the treatment preferences of physicians |
| Successful immune suppression followed by immune tolerance induction in a factor IX patient who had developed an anaphylactic reaction to factor IX | Mcsheffrey | 2012 | Full text not available |
| Review of the situation of diagnosis and treatment of inhibitors in patients with hemophilia in 13 Latin American countries | Montano | 2012 | Full text not available |
| Suppression of factor IX antibody in hemophilia B by factor IX and cyclophosphamide | Nilsson | 1973 | Case Report |
| Immunosuppressive treatment in haemophiliacs with inhibitors to factor VIII and factor IX | Nilsson | 1976 | Case Report |
| Induction of immune tolerance in hemophiliacs with inhibitors by combined treatment with i.v. IgG, cyclophosphamide and factor VIII or IX | Nilsson | 1990 | Full text not available |
| Management of haemophilia in Sweden | Nilsson | 1976 | Full text not available |
| Apheresis | Nilsson | 1995 | Full text not available |
| The Japanese Immune Tolerance Induction (J-ITI) study in haemophilia patients with inhibitor: Outcomes and successful predictors of ITI treatment | Nogami | 2018 | Excessive missing data and low quality of evidence |
| Resolution of nephrotic syndrome following rituximab therapy in a patient undergoing immune tolerance induction | Peltier | 2012 | Full text not available |
| Immunoadsorption for coagulation factor inhibitors: A retrospective critical appraisal of 10 consecutive cases from a single institution | Rivard | 2003 | The number of patients with hemophilia B is small |
| Natural history study of factor IX deficiency with focus on treatment and complications (B-Natural) | Shapiro | 2021 | The primary objectives of the study are the incidence of inhibitors in hemophilia B and joint complications |
| Inhibitors in hemophilia B: The Italian experience | Tagariello | 2011 | Duplicate publication of cohort Castaman et al. (2013)  Inhibitors in haemophilia B: The Italian experience |
| Factor IX inhibitors and anaphylaxis in hemophilia B | Warrier | 1997 | Duplicate publication of cohort Warrier et al. (2009)  Inhibitors in factor IX deficiency a report of the ISTH-SSC international FIX inhibitor registry (1997-200) |

**Supplementary Table S8.** Potential factors influencing ITI outcome

| **Variable** | **Study** | **Classification** | **Success** | **Failure** |
| --- | --- | --- | --- | --- |
| *F9* genotype | Li, 2022 | High risk | 8 | 7 |
|  |  | Low risk | 0 | 0 |
|  | Astermark, 2021 | High risk | 6 | 11 |
|  |  | Low risk | 7 | 8 |
|  | Castaman, 2013 | High risk | 5 | 0 |
|  |  | Low risk | 0 | 0 |
|  | Dou, 2022 | High risk | 2 | 5 |
|  |  | Low risk | 1 | 0 |
|  | Kihlberg, 2022 | High risk | 5 | 5 |
|  |  | Low risk | 3 | 6 |
| Interval between inhibitor development and ITI start | Li, 2022 | ≤1 month | 1 | 0 |
|  |  | >1 month | 7 | 7 |
|  | Astermark, 2021 | ≤1 month | 4 | 9 |
|  |  | >1 month | 7 | 10 |
|  | Freiburghaus, 1999 | ≤1 month | 0 | 0 |
|  |  | >1 month | 6 | 8 |
|  | Dou, 2022 | ≤1 month | 1 | 2 |
|  |  | >1 month | 2 | 7 |
|  | Kihlberg, 2022 | ≤1 month | 3 | 5 |
|  |  | >1 month | 5 | 6 |
| Interval between inhibitor development and ITI start | Li, 2022 | ≤12 months | 3 | 5 |
|  |  | >12 months | 0 | 7 |
|  | Astermark, 2021 | ≤12 months | 4 | 13 |
|  |  | >12 months | 9 | 4 |
|  | Freiburghaus, 1999 | ≤12 months | 0 | 0 |
|  |  | >12 months | 6 | 8 |
|  | Dou, 2022 | ≤12 months | 1 | 6 |
|  |  | >12 months | 2 | 3 |
|  | Kihlberg, 2022 | ≤12 months | 6 | 6 |
|  |  | >12 months | 2 | 5 |
| Interval between inhibitor development and ITI start | Li, 2022 | ≤24 months | 5 | 3 |
|  |  | >24 months | 3 | 4 |
|  | Astermark, 2021 | ≤24 months | 5 | 13 |
|  |  | >24 months | 8 | 4 |
|  | Freiburghaus, 1999 | ≤24 months | 0 | 0 |
|  |  | >24 months | 6 | 8 |
|  | Dou, 2022 | ≤24 months | 1 | 6 |
|  |  | >24 months | 2 | 3 |
|  | Kihlberg, 2022 | ≤24 months | 5 | 7 |
|  |  | >24 months | 3 | 4 |
| ITI product | Astermark, 2021 | pdFVIII | 8 | 10 |
|  |  | rFVIII | 5 | 8 |
|  | Freiburghaus, 1999 | pdFVIII | 6 | 8 |
|  |  | rFVIII | 0 | 0 |
|  | Dou, 2022 | pdFVIII | 3 | 10 |
|  |  | rFVIII | 0 | 0 |
|  | Kihlberg, 2022 | pdFVIII | 5 | 10 |
|  |  | rFVIII | 3 | 1 |
|  | Li, 2022 | pdFVIII | 8 | 7 |
|  |  | rFVIII | 0 | 0 |
| ITI dose | Li, 2022 | <100 IU/kg/day | 8 | 7 |
|  |  | ≥100 IU/kg/day | 0 | 0 |
|  | Astermark, 2021 | <100 IU/kg/day | 5 | 4 |
|  |  | ≥100 IU/kg/day | 8 | 18 |
|  | Freiburghaus, 1999 | <100 IU/kg/day | 2 | 1 |
|  |  | ≥100 IU/kg/day | 4 | 7 |
|  | Castaman, 2013 | <100 IU/kg/day | 4 | 4 |
|  |  | ≥100 IU/kg/day | 4 | 0 |
|  | Dou, 2022 | <100 IU/kg/day | 3 | 10 |
|  |  | ≥100 IU/kg/day | 0 | 0 |
|  | Kihlberg, 2022 | <100 IU/kg/day | 3 | 3 |
|  |  | ≥100 IU/kg/day | 5 | 6 |
| Immunosuppression therapy | Li, 2022 | Yes | 8 | 7 |
|  |  | No | 9 | 0 |
|  | Astermark, 2021 | Yes | 9 | 16 |
|  |  | No | 8 | 3 |
|  | Freiburghaus, 1999 | Yes | 6 | 8 |
|  |  | No | 0 | 0 |
|  | Dou, 2022 | Yes | 2 | 9 |
|  |  | No | 1 | 0 |
|  | Kihlberg, 2022 | Yes | 7 | 7 |
|  |  | No | 1 | 4 |
| Age at inhibitor development | Li, 2022 | ≤12 months | 0 | 0 |
|  |  | >12 months | 8 | 7 |
|  | Astermark, 2021 | ≤12 months | 0 | 4 |
|  |  | >12 months | 12 | 12 |
|  | Freiburghaus, 1999 | ≤12 months | 0 | 0 |
|  |  | >12 months | 6 | 8 |
|  | Castaman, 2013 | ≤12 months | 0 | 0 |
|  |  | >12 months | 5 | 0 |
|  | Dou, 2022 | ≤12 months | 0 | 0 |
|  |  | >12 months | 3 | 9 |
|  | Kihlberg, 2022 | ≤12 months | 0 | 0 |
|  |  | >12 months | 8 | 11 |
| Pre-ITI titer | Li, 2022 | <5 BU/mL | 0 | 0 |
|  |  | ≥5 BU/mL | 5 | 6 |
|  | Astermark, 2021 | <5 BU/mL | 10 | 13 |
|  |  | ≥5 BU/mL | 0 | 2 |
|  | Freiburghaus, 1999 | <5 BU/mL | 6 | 5 |
|  |  | ≥5 BU/mL | 0 | 3 |
|  | Dou, 2022 | <5 BU/mL | 0 | 0 |
|  |  | ≥5 BU/mL | 3 | 7 |
|  | Kihlberg, 2022 | <5 BU/mL | 7 | 5 |
|  |  | ≥5 BU/mL | 7 | 5 |
| Pre-ITI titer | Astermar 2021 | <10 BU/mL | 10 | 14 |
|  |  | ≥10 BU/mL | 0 | 1 |
|  | Dou 2022 | <10 BU/mL | 1 | 1 |
|  |  | ≥10 BU/mL | 2 | 6 |
|  | Freiburghaus 1999 | <10 BU/mL | 6 | 8 |
|  |  | ≥10 BU/mL | 0 | 0 |
|  | Kihlberg 2022 | <10 BU/mL | 7 | 8 |
|  |  | ≥10 BU/mL | 1 | 2 |
|  | Li 2022 | <10 BU/mL | 1 | 0 |
|  |  | ≥10 BU/mL | 4 | 6 |
| Historical peak titer | Li, 2022 | ≤100 BU/mL | 7 | 5 |
|  |  | >100 BU/mL | 1 | 1 |
|  | Astermark, 2021 | ≤100 BU/mL | 10 | 17 |
|  |  | >100 BU/mL | 1 | 1 |
|  | Freiburghaus, 1999 | ≤100 BU/mL | 1 | 0 |
|  |  | >100 BU/mL | 5 | 8 |
|  | Castaman, 2013 | ≤100 BU/mL | 4 | 1 |
|  |  | >100 BU/mL | 0 | 1 |
|  | Dou, 2022 | ≤100 BU/mL | 2 | 6 |
|  |  | >100 BU/mL | 1 | 1 |
|  | Kihlberg, 2022 | ≤100 BU/mL | 2 | 5 |
|  |  | >100 BU/mL | 1 | 1 |
| Historical peak titer | Li, 2022 | ≤200 BU/mL | 7 | 5 |
|  |  | >200 BU/mL | 1 | 21 |
|  | Astermark, 2021 | ≤200 BU/mL | 10 | 18 |
|  |  | >200 BU/mL | 1 | 0 |
|  | Freiburghaus, 1999 | ≤200 BU/mL | 3 | 2 |
|  |  | >200 BU/mL | 3 | 6 |
|  | Castaman, 2013 | ≤200 BU/mL | 4 | 1 |
|  |  | >200 BU/mL | 0 | 0 |
|  | Dou, 2022 | ≤200 BU/mL | 2 | 8 |
|  |  | >200 BU/mL | 1 | 1 |
|  | Kihlberg, 2022 | ≤200 BU/mL | 2 | 5 |
|  |  | >200 BU/mL | 1 | 1 |

Abbreviations: ITI = immune tolerance induction; High risk = large deletions and nonsense mutations; Low risk = small deletions or insertions, missense mutations, or splice site mutations; pdFVIII = plasma-derived FIX; rFVIII = recombinant FIX.
